# Supplementary material for: Selection and the direction of phenotypic evolution
Source: eLife. 2023 Aug 31;12:e80993. doi: 10.7554/eLife.80993 (PMC10564456; doi:10.7554/eLife.80993)
Supplement: Figure 1—source data 2. [file elife-80993-fig1-data2.pdf]

Number of phenotyped RILs per population in each environment

	High Salt	Low Salt
**A6140**	186	187
**GA150**	61	61
**GA250**	61	61
**GA450**	42	42

Raw output from R is available at:
https://github.com/ExpEvolWormLab/Mallard_Robertson/tree/main/output_files/txt/output_files/txt/sample_sizes_uniq_lines.txt
