# Supplementary material for: Selection and the direction of phenotypic evolution
Source: eLife. 2023 Aug 31;12:e80993. doi: 10.7554/eLife.80993 (PMC10564456; doi:10.7554/eLife.80993)
Supplement: Figure 1—source data 3. [file elife-80993-fig1-data3.pdf]

Factor	Df	Wilks	approx. F	numerator Df	denominator Df	Pr(>F)
salt environment	1	0.13728	523.41	7	583.0	< 2.2e-16
population	3	0.30973	40.20	21	1674.6	< 2.2e-16
temperature	1	0.52131	76.48	7	583.0	< 2.2e-16
humidity	1	0.98677	1.12	7	583.0	0.3508
log density	1	0.26888	226.47	7	583.0	< 2.2e-16
Assay year	1	0.58612	58.81	7	583.0	< 2.2e-16
block effect	96	0.17860	1.70	672	4085.4	< 2.2e-16
salt environment x population	3	0.82841	5.40	21	1674.6	3.99e-14
temperature x humidity	1	0.98433	1.33	7	583.0	0.2352
temperature x log density	1	0.93379	5.91	7	583.0	1.20e-06
humidity x log density	1	0.99185	0.68	7	583.0	0.6856
temperature x humidity x log density	1	0.98383	1.37	7	583.0	0.2160
Residuals	589					

Raw output from R is available at: https://github.com/ExpEvolWormLab/Mallard_Robertson/tree/main/output_files/txt/output_files/txt/Manova_results.txt
