# Supplementary material for: Selection and the direction of phenotypic evolution
Source: eLife. 2023 Aug 31;12:e80993. doi: 10.7554/eLife.80993 (PMC10564456; doi:10.7554/eLife.80993)
Supplement: Figure 1—source data 4. [file elife-80993-fig1-data4.pdf]

Trait : SF

	Df	Sum Sq	Mean Sq	F value	Pr (>F)	Sign.
salt environment	1	17.716	17.716	75.8635	< 2.2e-16	***
population	3	28.261	9.420	40.3404	< 2.2e-16	***
temperature	1	49.034	49.034	209.9752	< 2.2e-16	***
humidity	1	0.078	0.078	0.3343	0.5633749	
log density	1	72.404	72.404	310.0496	< 2.2e-16	***
Assay year	1	10.868	10.868	46.5368	2.236e-11	***
block effect	96	41.571	0.433	1.8543	8.663e-06	***
salt environment x population	3	4.539	1.513	6.4796	0.0002556	***
temperature x humidity	1	0.197	0.197	0.8427	0.3590055	
temperature x log density	1	0.000	0.000	0.0002	0.9892668	
humidity x log density	1	0.028	0.028	0.1183	0.7309616	
temperature x humidity x log density	1	0.021	0.021	0.0897	0.7646773	
Residuals	589	137.546	0.234			

Trait : SF

	Df	Sum Sq	Mean Sq	F value	Pr (>F)	Sign.
salt environment	1	1.200	1.200	2.4854	0.11544	
population	3	135.427	45.142	93.5105	< 2.2e-16	***
temperature	1	1.436	1.436	2.9744	0.08512	.
humidity	1	2.136	2.136	4.4240	0.03586	*
log density	1	187.535	187.535	388.4689	< 2.2e-16	***
Assay year	1	0.128	0.128	0.2647	0.60710	
block effect	96	103.359	1.077	2.2302	6.572e-09	***
salt environment x population	3	0.757	0.252	0.5230	0.66665	
temperature x humidity	1	0.688	0.688	1.4252	0.23304	
temperature x log density	1	3.092	3.092	6.4052	0.01164	*
humidity x log density	1	0.141	0.141	0.2913	0.58959	
temperature x humidity x log density	1	2.640	2.640	5.4693	0.01969	*
Residuals	589	284.342	0.483			

Trait : SB

	Df	Sum Sq	Mean Sq	F value	Pr (>F)	Sign.
salt environment	1	8.519	8.519	29.1247	9.849e-08	***
population	3	70.659	23.553	80.5179	< 2.2e-16	***
temperature	1	69.665	69.665	238.1569	< 2.2e-16	***
humidity	1	0.252	0.252	0.8627	0.3533686	
log density	1	107.195	107.195	366.4568	< 2.2e-16	***
Assay year	1	21.361	21.361	73.0238	< 2.2e-16	***
block effect	96	46.770	0.487	1.6655	0.0002212	***
salt environment x population	3	6.447	2.149	7.3460	7.698e-05	***
temperature x humidity	1	0.012	0.012	0.0420	0.8377404	
temperature x log density	1	1.128	1.128	3.8551	0.0500663	.
humidity x log density	1	0.005	0.005	0.0168	0.8969976	
temperature x humidity x log density	1	0.233	0.233	0.7976	0.3721879	
Residuals	589	172.293	0.293			

Trait : BS

	Df	Sum Sq	Mean Sq	F value	Pr (>F)	Sign.
salt environment	1	7.167	7.167	136.2041	< 2.2e-16	***
population	3	32.594	10.865	206.4898	< 2.2e-16	***
temperature	1	7.736	7.736	147.0326	< 2.2e-16	***
humidity	1	0.032	0.032	0.6176	0.4322598	
log density	1	58.431	58.431	1110.5059	< 2.2e-16	***
Assay year	1	4.550	4.550	86.4687	< 2.2e-16	***
block effect	96	7.960	0.083	1.5758	0.0009149	***
salt environment x population	3	0.065	0.022	0.4123	0.7442336	
temperature x humidity	1	0.035	0.035	0.6653	0.4150272	
temperature x log density	1	0.124	0.124	2.3536	0.1255323	
humidity x log density	1	0.039	0.039	0.7480	0.3874554	
temperature x humidity x log density	1	0.011	0.011	0.2125	0.6450113	
Residuals	589	30.991	0.053			

Trait : FS

	Df	Sum Sq	Mean Sq	F value	Pr (>F)	Sign.
salt environment	1	20.891	20.891	190.9788	< 2.2e-16	***
population	3	39.981	13.327	121.8293	< 2.2e-16	***
temperature	1	9.531	9.531	87.1315	< 2.2e-16	
humidity	1	0.171	0.171	1.5601	0.2121506	***
log density	1	124.781	124.781	140.6942	< 2.2e-16	***
Assay year	1	6.086	6.086	55.6380	3.138e-13	**
block effect	96	16.155	0.168	1.5384	0.0016106	***
salt environment x population	3	1.884	0.628	5.7405	0.0007105	
temperature x humidity	1	0.005	0.005	0.0458	0.8305695	*
temperature x log density	1	0.664	0.664	6.0734	0.0140079	
humidity x log density	1	0.180	0.180	1.6430	0.2004148	
temperature x humidity x log density	1	0.009	0.009	0.0778	0.7804465	
Residuals	589	64.431	0.109			

Trait : BF

	Df	Sum Sq	Mean Sq	F value	Pr (>F)	Sign.
salt environment	1	3.098	3.098	6.4474	0.01137	*
population	3	110.036	36.679	76.3437	< 2.2e-16	***
temperature	1	0.483	0.483	1.0049	0.31654	
humidity	1	2.857	2.857	5.9467	0.01504	*
log density	1	118.889	118.889	247.4584	< 2.2e-16	***
Assay year	1	0.663	0.663	1.3794	0.24067	
block effect	96	101.729	1.060	2.2056	1.076e-08	***
salt environment x population	3	1.607	0.536	1.1152	0.34225	
temperature x humidity	1	1.006	1.006	2.0934	0.14847	
temperature x log density	1	11.968	11.968	24.9112	7.916e-07	***
humidity x log density	1	0.094	0.094	0.1956	0.65847	
temperature x humidity x log density	1	2.963	2.963	6.1678	0.01329	*
Residuals	589	282.980	0.480			

Trait : Size

	Df	Sum Sq	Mean Sq	F value	Pr (>F)	Sign.
salt environment	1	746.97	746.97	2228.2075	< 2.2e-16	***
population	3	88.79	29.60	88.2885	< 2.2e-16	***
temperature	1	58.52	58.52	174.5597	< 2.2e-16	***
humidity	1	0.00	0.00	0.0007	0.979245	
log density	1	31.91	31.91	95.1985	< 2.2e-16	***
Assay year	1	83.38	83.38	248.7218	< 2.2e-16	***
block effect	96	45.52	0.47	1.4143	0.009258	**
salt environment x population	3	19.48	6.49	19.3727	5.442e-12	***
temperature x humidity	1	1.53	1.53	4.5585	0.033168	*
temperature x log density	1	0.08	0.08	0.2422	0.622780	
humidity x log density	1	0.01	0.01	0.0210	0.884725	
temperature x humidity x log density	1	0.09	0.09	0.2740	0.600842	
Residuals	589	197.45	0.34			

Signif. codes: 0 ‘***’ 0.001 ‘**’ 0.01 ‘*’ 0.05 ‘.’ 0.1 ‘ ’ 1

Raw output from R is available at:
https://github.com/ExpEvolWormLab/Mallard_Robertson/tree/main/output_files/txt/Manova_trait_responses.txt
