# Supplementary material for: Selection and the direction of phenotypic evolution
Source: eLife. 2023 Aug 31;12:e80993. doi: 10.7554/eLife.80993 (PMC10564456; doi:10.7554/eLife.80993)
Supplement: Figure 1—source data 5. [file elife-80993-fig1-data5.pdf]

Eigenvalues:	805.5	2.2 e-13	9.0 e-15	1.26 e-17	-5 e16	-8.7 e-15	-1.1 e-14
**Loadings:**							
	**pmax**	**p2**	**p3**	**p4**	**p5**	**p6**	**p7**
**SF**	0.148	0.989	0	0	0	0	0
**SB**	-0.103	0.015	0.912	0.003	0.003	-0.307	0.253
**FS**	0.161	-0.024	-0.375	-0.066	0.097	-0.746	0.512
**FB**	-0.039	0.006	0.032	-0.966	0.241	0.044	-0.07
**BS**	0.094	-0.014	0.042	-0.008	-0.151	-0.569	-0.801
**BF**	0.062	-0.009	-0.022	-0.251	-0.951	0.036	0.163
**Size**	-0.963	0.144	-0.159	0.01	-0.07	-0.147	-0.007

Raw output from R is available at:
https://github.com/ExpEvolWormLab/Mallard_Robertson/tree/main/output_files/txt/SSCP_plasticity_ED.txt
