# Supplementary material for: Selection and the direction of phenotypic evolution
Source: eLife. 2023 Aug 31;12:e80993. doi: 10.7554/eLife.80993 (PMC10564456; doi:10.7554/eLife.80993)
Supplement: Figure 1—figure supplement 1—source data 1. [file elife-80993-fig1-figsupp1-data1.pdf]

contrast	trait	estimate	SE	df	t.ratio	p.value
A6140: High Salt - Low Salt	SF	0.188	0.037	662.457	5.062	1.5 e-05
A6140: High Salt - Low Salt	SB	-0.194	0.041	661.29	-4.684	9.3 e-05
A6140: High Salt - Low Salt	FS	0.212	0.025	670.405	8.449	< 1 e-10
A6140: High Salt - Low Salt	FB	-0.152	0.055	673.651	-2.756	0.11
A6140: High Salt - Low Salt	BS	0.083	0.017	670.494	4.762	6.4 e-05
A6140: High Salt - Low Salt	BF	0.026	0.055	666.402	0.464	0.99
A6140: High Salt - Low Salt	Size	-1.234	0.044	682.086	-27.835	< 1 e-10

Raw output from R is available at:
https://github.com/ExpEvolWormLab/Mallard_Robertson/blob/main/output_files/txt/Plasticity_contrasts.txt
