# Supplementary material for: Selection and the direction of phenotypic evolution
Source: eLife. 2023 Aug 31;12:e80993. doi: 10.7554/eLife.80993 (PMC10564456; doi:10.7554/eLife.80993)
Supplement: Table 3—source data 2. [file elife-80993-table3-data2.pdf]

| | **A6**140 - Low Salt | | | | | | | **A6**140 - High Salt | | | | | | |
|---|---|---|---|---|---|---|---|---|---|---|---|---|---|---|
| | $g_{max}$ | $g_2$ | $g_3$ | $g_4$ | $g_5$ | $g_6$ | g7 | $g_{max}$ | $g_2$ | $g_3$ | $g_4$ | $g_5$ | $g_6$ | g7 |
| Eigenvalues | 0.356 | 0.113 | 0.05 | 0.02 | 0.009 | 0.005 | 0.003 | 0.488 | 0.06 | 0.039 | 0.03 | 0.014 | 0.008 | 0.005 |
| HPD lower | 0.257 | 0.078 | 0.036 | 0.015 | 0.007 | 0.004 | 0.002 | 0.271 | 0.046 | 0.03 | 0.022 | 0.011 | 0.007 | 0.004 |
| HPD upper | 0.449 | 0.156 | 0.068 | 0.029 | 0.014 | 0.006 | 0.004 | 0.697 | 0.09 | 0.052 | 0.038 | 0.02 | 0.011 | 0.007 |
| Proportion | 0.64 | 0.203 | 0.09 | 0.036 | 0.016 | 0.009 | 0.005 | 0.758 | 0.093 | 0.061 | 0.047 | 0.022 | 0.012 | 0.008 |
| *Trait loadings:* | | | | | | | | | | | | | | |
| SF | -0.334 | 0.554 | 0.097 | -0.004 | 0.595 | 0.467 | 0 | -0.36 | -0.388 | 0.241 | 0.283 | -0.466 | 0.58 | 0.165 |
| SB | -0.32 | 0.506 | 0.087 | 0.541 | -0.252 | -0.521 | -0.083 | -0.459 | -0.409 | 0.394 | -0.186 | -0.057 | -0.611 | -0.234 |
| FS | 0.179 | -0.334 | 0.076 | 0.389 | 0.548 | -0.186 | -0.603 | 0.267 | 0.303 | 0.129 | 0.24 | -0.693 | -0.443 | 0.296 |
| FB | 0.568 | 0.208 | -0.419 | 0.543 | -0.087 | 0.362 | 0.16 | 0.532 | -0.502 | -0.117 | -0.585 | -0.316 | 0.088 | -0.05 |
| BS | 0.107 | -0.125 | 0.079 | 0.12 | 0.48 | -0.401 | 0.749 | 0.142 | 0.153 | 0.035 | 0.225 | -0.257 | 0.118 | -0.908 |
| BF | 0.396 | 0.461 | -0.36 | -0.495 | 0.182 | -0.425 | -0.207 | 0.467 | -0.496 | 0.15 | 0.619 | 0.323 | -0.157 | 0.038 |
| Size | -0.513 | -0.234 | -0.816 | 0.045 | 0.106 | -0.055 | 0.017 | -0.258 | -0.264 | -0.856 | 0.227 | -0.173 | -0.22 | -0.041 |

**Raw output from R is available at:** https://github.com/ExpEvolWormLab/Mallard_Robertson/tree/main/output_files/txt/output_files/G_matrix_eigendecomposition/
