# Supplementary material for: Selection and the direction of phenotypic evolution
Source: eLife. 2023 Aug 31;12:e80993. doi: 10.7554/eLife.80993 (PMC10564456; doi:10.7554/eLife.80993)
Supplement: Table 3—source data 3. [file elife-80993-table3-data3.pdf]

Eigenvalues:	483	15.4	7.5	8 e-15	7 e-15	-7 e-15	-2 e-14
**Loadings:**							
	**dmax**	**d2**	**d3**	**d4**	**d5**	**d6**	**d7**
**SF**	-0.225	-0.253	0.62	0	0	0	0.708
**SB**	-0.365	-0.616	0.282	0.186	-0.122	-0.142	-0.582
**FS**	0.284	0.155	0.28	0.747	0.484	0.121	-0.099
**FB**	0.517	-0.578	-0.38	0.271	-0.198	-0.241	0.29
**BS**	0.253	0.26	0.31	0.214	-0.817	0.223	-0.098
**BF**	0.473	-0.33	0.226	-0.441	0.195	0.598	-0.165
**Size**	-0.425	-0.15	-0.409	0.306	-0.081	0.707	0.17

Raw output from R is available at:
https://github.com/ExpEvolWormLab/Mallard_Robertson/tree/main/output_files/txt/SSCP_divergence_ED.txt
