# Supplementary material for: Selection and the direction of phenotypic evolution
Source: eLife. 2023 Aug 31;12:e80993. doi: 10.7554/eLife.80993 (PMC10564456; doi:10.7554/eLife.80993)
Supplement: Table 3—source data 4. [file elife-80993-table3-data4.pdf]

	83% CI		95% CI		posterior
**Trait**	lower	upper	lower	upper	mode
SF	-1.43	-0.29	-1.76	0.05	-0.93
SB	0.36	1.43	0.12	1.79	0.931
FS	-0.34	1.09	-0.82	1.42	0.353
FB	-0.12	0.73	-0.36	0.93	0.328
BS	-2.22	-0.11	-2.87	0.46	-0.963
BF	-1.27	-0.36	-1.61	-0.1	-0.824
Size	0.36	1.4	-0.01	1.67	0.834

Raw output from R is available at:
https://github.com/ExpEvolWormLab/Mallard_Robertson/blob/main/output_files/txt/Selection_gradients.txt
