# Supplementary material for: Selection and the direction of phenotypic evolution
Source: eLife. 2023 Aug 31;12:e80993. doi: 10.7554/eLife.80993 (PMC10564456; doi:10.7554/eLife.80993)
Supplement: Table 3—source data 5. [file elife-80993-table3-data5.pdf]

**MA lines**- Low Salt							
	m_{max}	m_2	m_3	m_4	m_5	m_6	m7
Eigenvalues	0.31	0.29	0.06	0.05	0.04	0.02	0.009
Proportion	0.39	0.37	0.08	0.06	0.05	0.03	0.01
Trait loadings:							
SF	-0.4	-0.13	-0.58	0.22	-0.09	0.47	0.45
SB	-0.22	-0.21	-0.42	0.39	-0.23	-0.46	-0.56
FS	0.61	0.26	-0.28	0.37	0.25	0.43	-0.32
FB	0.63	-0.52	-0.24	-0.17	-0.36	-0.17	0.29
BS	0.08	0.11	-0.29	0.09	0.67	-0.54	0.37
BF	-0.1	-0.76	0.18	0.01	0.52	0.24	-0.21
Size	0.07	-0.1	0.48	0.79	-0.14	-0.08	0.32

Raw output from R is available at:

https://github.com/ExpEvolWormLab/Mallard_Robertson/blob/main/output_files/txt/MAlines_eigenvectors.txt
