# Supplementary material for: Selection and the direction of phenotypic evolution
Source: eLife. 2023 Aug 31;12:e80993. doi: 10.7554/eLife.80993 (PMC10564456; doi:10.7554/eLife.80993)
Supplement: Figure 2—source data 1. [file elife-80993-fig2-data1.pdf]

A6140 in low salt

	SF	SB	FS	FB	BS	BF	Size
**SF**	**0.078 [0.054;0.102]**	0.064 [0.043;0.09]	-0.037 [-0.052;-0.026]	-0.051 [-0.082;-0.034]	-0.017 [-0.027;-0.011]	-0.017 [-0.037;0.002]	0.038 [0.015;0.06]
**SB**	0.064 [0.043;0.09]	**0.071 [0.05;0.102]**	-0.035 [-0.048;-0.022]	-0.046 [-0.073;-0.026]	-0.017 [-0.028;-0.011]	-0.021 [-0.045;-0.005]	0.038 [0.015;0.061]
**FS**	-0.037 [-0.052;-0.026]	-0.035 [-0.048;-0.022]	**0.031 [0.022;0.041]**	0.026 [0.013;0.042]	0.015 [0.009;0.021]	0.003 [-0.007;0.017]	-0.024 [-0.038;-0.011]
**FB**	-0.051 [-0.082;-0.034]	-0.046 [-0.073;-0.026]	0.026 [0.013;0.042]	**0.127 [0.096;0.172]**	0.015 [0.006;0.026]	0.087 [0.059;0.12]	-0.084 [-0.12;-0.059]
**BS**	-0.017 [-0.027;-0.011]	-0.017 [-0.028;-0.011]	0.015 [0.009;0.021]	0.015 [0.006;0.026]	**0.012 [0.008;0.016]**	0.007 [-0.002;0.014]	-0.017 [-0.028;-0.01]
**BF**	-0.017 [-0.037;0.002]	-0.021 [-0.045;-0.005]	0.003 [-0.007;0.017]	0.087 [0.059;0.12]	0.007 [-0.002;0.014]	**0.089 [0.066;0.128]**	-0.064 [-0.096;-0.043]
**Size**	0.038 [0.015;0.06]	0.038 [0.015;0.061]	-0.024 [-0.038;-0.011]	-0.084 [-0.12;-0.059]	-0.017 [-0.028;-0.01]	-0.064 [-0.096;-0.043]	**0.128 [0.098;0.169]**

A6140 in high salt

	SF	SB	FS	FB	BS	BF	Size
**SF**	**0.08 [0.051;0.128]**	0.084 [0.042;0.134]	-0.045 [-0.075;-0.024]	-0.079 [-0.127;-0.041]	-0.023 [-0.039;-0.01]	-0.063 [-0.11;-0.028]	0.043 [0.02;0.069]
**SB**	0.084 [0.042;0.134]	**0.117 [0.069;0.19]**	-0.06 [-0.101;-0.034]	-0.098 [-0.144;-0.038]	-0.033 [-0.052;-0.015]	-0.087 [-0.149;-0.043]	0.049 [0.024;0.086]
**FS**	-0.045 [-0.075;-0.024]	-0.06 [-0.101;-0.034]	**0.052 [0.032;0.074]**	0.055 [0.028;0.091]	0.023 [0.013;0.037]	0.05 [0.027;0.089]	-0.036 [-0.057;-0.02]
**FB**	-0.079 [-0.127;-0.041]	-0.098 [-0.144;-0.038]	0.055 [0.028;0.091]	**0.156 [0.095;0.214]**	0.028 [0.012;0.048]	0.116 [0.067;0.178]	-0.054 [-0.087;-0.029]
**BS**	-0.023 [-0.039;-0.01]	-0.033 [-0.052;-0.015]	0.023 [0.013;0.037]	0.028 [0.012;0.048]	**0.019 [0.012;0.029]**	0.028 [0.013;0.048]	-0.018 [-0.031;-0.009]
**BF**	-0.063 [-0.11;-0.028]	-0.087 [-0.149;-0.043]	0.05 [0.027;0.089]	0.116 [0.067;0.178]	0.028 [0.013;0.048]	**0.128 [0.083;0.198]**	-0.049 [-0.08;-0.024]
**Size**	0.043 [0.02;0.069]	0.049 [0.024;0.086]	-0.036 [-0.057;-0.02]	-0.054 [-0.087;-0.029]	-0.018 [-0.031;-0.009]	-0.049 [-0.08;-0.024]	**0.063 [0.046;0.091]**

Raw output from R is available at: https://github.com/ExpEvolWormLab/Mallard_Robertson/tree/main/output_files/txt/output_files/G_mat_tables/
