# Supplementary material for: Selection and the direction of phenotypic evolution
Source: eLife. 2023 Aug 31;12:e80993. doi: 10.7554/eLife.80993 (PMC10564456; doi:10.7554/eLife.80993)
Supplement: Figure 3—source data 1. [file elife-80993-fig3-data1.pdf]

**Figure 3A:**

**Null**

| | |
|---|---|
| 95% CI | [0.13 - 0.28] |

**Observed**

| | |
|---|---|
| Mean | 0.17 |
| 83% CI | [0.13 - 0.22] |
| 95% CI | [0.11 - 0.24] |

**Figure 3B:**

**Null**

| | |
|---|---|
| 83% CI | [57.5 - 90.0] |
| 95% CI | [45.8 - 90.0] |

**Observed**

| | gmax | g2 | g3 |
|---|---|---|---|
| Mean | 71.7 | 74.2 | 51.4 |
| 83% CI | [67.6 - 75.0] | [64.1 - 90.0] | [22.3 - 74.8] |
| 95% CI | [66.5 - 72.3] | [54.1 - 90.0] | [23.2 - 87.6] |
