# Supplementary material for: Selection and the direction of phenotypic evolution
Source: eLife. 2023 Aug 31;12:e80993. doi: 10.7554/eLife.80993 (PMC10564456; doi:10.7554/eLife.80993)
Supplement: Figure 4—source data 1. [file elife-80993-fig4-data1.pdf]

Trait	Environment	Posterior Mode	83% CI		95% CI	
			lower	upper	lower	upper
SF*w	High Salt	0.035	0.018	0.06	0.011	0.073
SB*w	High Salt	0.073	0.042	0.099	0.032	0.113
FS*w	High Salt	-0.044	-0.062	-0.027	-0.072	-0.023
FB*w	High Salt	-0.055	-0.087	-0.033	-0.101	-0.023
BS*w	High Salt	-0.03	-0.042	-0.02	-0.049	-0.016
BF*w	High Salt	-0.076	-0.099	-0.045	-0.115	-0.035
Size*w	High Salt	0.05	0.034	0.068	0.028	0.077
w	High Salt	0.22	0.188	0.255	0.176	0.271
SF*w	Low Salt	0.002	-0.012	0.022	-0.02	0.03
SB*w	Low Salt	0.017	-0.002	0.034	-0.007	0.044
FS*w	Low Salt	-0.014	-0.025	-0.003	-0.032	0.001
FB*w	Low Salt	-0.031	-0.049	-0.005	-0.058	0.006
BS*w	Low Salt	-0.011	-0.02	-0.004	-0.024	-0.001
BF*w	Low Salt	-0.026	-0.05	-0.01	-0.057	6 e-5
Size*w	Low Salt	0.065	0.051	0.095	0.044	0.108

Raw output from R is available at:
https://github.com/ExpEvolWormLab/Mallard_Robertson/blob/main/output_files/txt/Selection_differentials.txt
