# Supplementary material for: Selection and the direction of phenotypic evolution
Source: eLife. 2023 Aug 31;12:e80993. doi: 10.7554/eLife.80993 (PMC10564456; doi:10.7554/eLife.80993)
Supplement: Figure 6—source data 2. [file elife-80993-fig6-data2.pdf]

High Salt

contrast	trait	estimate	SE	df	t.ratio	p.value
A6140 - GA150	SF	-0.03	0.054	687.668	-0.568	0.99
A6140 - GA250	SF	-0.252	0.056	735.093	-4.505	2.1 e-05
A6140 - GA450	SF	-0.05	0.062	684.893	-0.804	0.99
A6140 - GA150	SB	-0.148	0.06	687.718	-2.475	0.21
A6140 - GA250	SB	-0.317	0.063	733.219	-5.055	1.5 e-05
A6140 - GA450	SB	-0.294	0.069	683.122	-4.227	7.0 e-04
A6140 - GA150	FS	0.117	0.036	692.681	3.241	0.027
A6140 - GA250	FS	0.129	0.038	741.552	3.436	0.014
A6140 - GA450	FS	0.143	0.042	691.12	3.407	0.015
A6140 - GA150	FB	0.277	0.08	694.743	3.481	0.012
A6140 - GA250	FB	0.441	0.083	743.203	5.338	3.5 e-06
A6140 - GA450	FB	0.125	0.092	693.096	1.351	0.88
A6140 - GA150	BS	0.09	0.025	692.857	3.557	9.5 e-03
A6140 - GA250	BS	0.117	0.026	741.393	4.472	2.4 e-04
A6140 - GA450	BS	0.147	0.029	691.012	5.026	1.8 e-05
A6140 - GA150	BF	0.383	0.079	691.011	4.823	4.7 e-05
A6140 - GA250	BF	0.341	0.083	737.288	4.12	0.0011
A6140 - GA450	BF	0.297	0.092	687.091	3.23	0.028
A6140 - GA150	Size	-0.051	0.064	698.9	-0.804	0.99
A6140 - GA250	Size	-0.204	0.066	746.862	-3.099	0.042
A6140 - GA450	Size	-0.078	0.074	698.483	-1.058	0.96

Raw output from R is available at:
https://github.com/ExpEvolWormLab/Mallard_Robertson/blob/main/output_files/txt/Divergence_contrasts_High_Salt.txt
