# Supplementary material for: Selection and the direction of phenotypic evolution
Source: eLife. 2023 Aug 31;12:e80993. doi: 10.7554/eLife.80993 (PMC10564456; doi:10.7554/eLife.80993)
Supplement: Figure 6—figure supplement 2—source data 1. [file elife-80993-fig6-figsupp2-data1.pdf]

Low Salt

contrast	trait	estimate	SE	df	t.ratio	p.value
A6140 - GA150	SF	0.24	0.054	693.317	4.473	2.4 e-04
A6140 - GA250	SF	0.004	0.054	694.498	0.071	0.99
A6140 - GA450	SF	0.104	0.062	686.124	1.678	0.70
A6140 - GA150	SB	0.206	0.06	694.078	3.447	0.01
A6140 - GA250	SB	-0.118	0.06	693.758	-1.962	0.51
A6140 - GA450	SB	-0.054	0.069	684.824	-0.776	0.99
A6140 - GA150	FS	-0.012	0.036	696.53	-0.334	0.99
A6140 - GA250	FS	-0.037	0.036	698.661	-1.02	0.97
A6140 - GA450	FS	-0.005	0.042	691.17	-0.123	0.99
A6140 - GA150	FB	0.252	0.079	698.075	3.174	0.034
A6140 - GA250	FB	0.375	0.08	699.836	4.711	8.1 e-05
A6140 - GA450	FB	0.003	0.092	692.826	0.033	0.99
A6140 - GA150	BS	0.072	0.025	696.808	2.853	0.084
A6140 - GA250	BS	0.08	0.025	698.612	3.18	0.032
A6140 - GA450	BS	0.15	0.029	691.145	5.139	9.9 e-06
A6140 - GA150	BF	0.297	0.079	696.378	3.749	0.0047
A6140 - GA250	BF	0.379	0.079	696.332	4.766	6.2 e-05
A6140 - GA450	BF	0.129	0.092	688.209	1.406	0.85
A6140 - GA150	Size	-0.45	0.063	700.232	-7.093	0
A6140 - GA250	Size	-0.707	0.064	702.494	-11.106	0
A6140 - GA450	Size	-0.573	0.074	696.848	-7.783	0

Raw output from R is available at:
https://github.com/ExpEvolWormLab/Mallard_Robertson/blob/main/output_files/txt/Divergence_contrasts_Low_Salt.txt
