# Supplementary material for: Selection and the direction of phenotypic evolution
Source: eLife. 2023 Aug 31;12:e80993. doi: 10.7554/eLife.80993 (PMC10564456; doi:10.7554/eLife.80993)
Supplement: Figure 7—source data 1. [file elife-80993-fig7-data1.pdf]

GA150 in low salt

	SF	SB	FS	FB	BS	BF	Size
**SF**	**0.029 [0.015;0.064]**	0.024 [0.005;0.056]	-0.002 [-0.016;0.005]	-0.003 [-0.034;0.022]	-0.002 [-0.013;0.005]	0.003 [-0.021;0.031]	0.008 [-0.015;0.038]
**SB**	0.024 [0.005;0.056]	**0.039 [0.019;0.079]**	-0.006 [-0.02;0.005]	0.003 [-0.03;0.033]	-0.004 [-0.016;0.003]	0.003 [-0.021;0.038]	0.006 [-0.017;0.042]
**FS**	-0.002 [-0.016;0.005]	-0.006 [-0.02;0.005]	**0.01 [0.006;0.021]**	-0.009 [-0.022;0.008]	0.005 [0;0.011]	-0.008 [-0.023;0.006]	0.004 [-0.015;0.016]
**FB**	-0.003 [-0.034;0.022]	0.003 [-0.03;0.033]	-0.009 [-0.022;0.008]	**0.073 [0.037;0.147]**	-0.004 [-0.017;0.008]	0.047 [0.015;0.108]	-0.027 [-0.077;0.005]
**BS**	-0.002 [-0.013;0.005]	-0.004 [-0.016;0.003]	0.005 [0;0.011]	-0.004 [-0.017;0.008]	**0.007 [0.004;0.013]**	-0.004 [-0.015;0.008]	-0.003 [-0.016;0.008]
**BF**	0.003 [-0.021;0.031]	0.003 [-0.021;0.038]	-0.008 [-0.023;0.006]	0.047 [0.015;0.108]	-0.004 [-0.015;0.008]	**0.061 [0.03;0.124]**	-0.024 [-0.064;0.01]
**Size**	0.008 [-0.015;0.038]	0.006 [-0.017;0.042]	0.004 [-0.015;0.016]	-0.027 [-0.077;0.005]	-0.003 [-0.016;0.008]	-0.024 [-0.064;0.01]	**0.067 [0.034;0.134]**

GA150 in high salt

	SF	SB	FS	FB	BS	BF	Size
**SF**	**0.04 [0.018;0.07]**	0.027 [0.007;0.059]	-0.011 [-0.027;-0.001]	-0.018 [-0.048;0.006]	-0.002 [-0.016;0.003]	0.001 [-0.02;0.022]	0.023 [0.002;0.056]
**SB**	0.027 [0.007;0.059]	**0.048 [0.024;0.089]**	-0.013 [-0.031;-0.001]	0.005 [-0.026;0.032]	-0.009 [-0.024;-0.001]	0.003 [-0.022;0.026]	0.021 [-0.003;0.055]
**FS**	-0.011 [-0.027;-0.001]	-0.013 [-0.031;-0.001]	**0.015 [0.008;0.028]**	0.005 [-0.011;0.024]	0.005 [0.001;0.014]	-0.002 [-0.014;0.013]	-0.009 [-0.031;0.002]
**FB**	-0.018 [-0.048;0.006]	0.005 [-0.026;0.032]	0.005 [-0.011;0.024]	**0.085 [0.048;0.142]**	-0.001 [-0.014;0.011]	0.034 [0.009;0.076]	-0.025 [-0.058;0.005]
**BS**	-0.002 [-0.016;0.003]	-0.009 [-0.024;-0.001]	0.005 [0.001;0.014]	-0.001 [-0.014;0.011]	**0.01 [0.006;0.018]**	0.001 [-0.009;0.013]	-0.008 [-0.023;0.002]
**BF**	0.001 [-0.02;0.022]	0.003 [-0.022;0.026]	-0.002 [-0.014;0.013]	0.034 [0.009;0.076]	0.001 [-0.009;0.013]	**0.047 [0.028;0.093]**	-0.003 [-0.032;0.021]
**Size**	0.023 [0.002;0.056]	0.021 [-0.003;0.055]	-0.009 [-0.031;0.002]	-0.025 [-0.058;0.005]	-0.008 [-0.023;0.002]	-0.003 [-0.032;0.021]	**0.06 [0.032;0.111]**

GA250 in low salt

	SF	SB	FS	FB	BS	BF	Size
**SF**	**0.045 [0.02;0.081]**	0.041 [0.015;0.079]	-0.007 [-0.023;0.005]	-0.001 [-0.028;0.022]	-0.004 [-0.017;0.003]	0.006 [-0.018;0.039]	0.024 [-0.003;0.063]
**SB**	0.041 [0.015;0.079]	**0.054 [0.028;0.101]**	-0.005 [-0.022;0.008]	0.002 [-0.025;0.03]	-0.007 [-0.019;0.003]	0.003 [-0.023;0.04]	0.034 [0;0.073]
**FS**	-0.007 [-0.023;0.005]	-0.005 [-0.022;0.008]	**0.016 [0.009;0.03]**	-0.003 [-0.017;0.013]	0.008 [0.002;0.015]	-0.01 [-0.03;0.004]	-0.01 [-0.034;0.006]
**FB**	-0.001 [-0.028;0.022]	0.002 [-0.025;0.03]	-0.003 [-0.017;0.013]	**0.05 [0.024;0.093]**	-0.002 [-0.015;0.007]	0.029 [0.002;0.07]	-0.02 [-0.064;0.01]
**BS**	-0.004 [-0.017;0.003]	-0.007 [-0.019;0.003]	0.008 [0.002;0.015]	-0.002 [-0.015;0.007]	**0.009 [0.005;0.016]**	-0.005 [-0.018;0.006]	-0.011 [-0.026;0.003]
**BF**	0.006 [-0.018;0.039]	0.003 [-0.023;0.04]	-0.01 [-0.03;0.004]	0.029 [0.002;0.07]	-0.005 [-0.018;0.006]	**0.056 [0.027;0.111]**	-0.019 [-0.059;0.02]
**Size**	0.024 [-0.003;0.063]	0.034 [0;0.073]	-0.01 [-0.034;0.006]	-0.02 [-0.064;0.01]	-0.011 [-0.026;0.003]	-0.019 [-0.059;0.02]	**0.098 [0.058;0.185]**

GA250 in high salt

	SF	SB	FS	FB	BS	BF	Size
**SF**	**0.03 [0.014;0.066]**	0.015 [0.002;0.049]	-0.003 [-0.022;0.006]	-0.019 [-0.044;0.005]	-0.001 [-0.013;0.008]	0.002 [-0.021;0.028]	0.012 [-0.012;0.041]
**SB**	0.015 [0.002;0.049]	**0.034 [0.015;0.068]**	-0.009 [-0.026;0.003]	-0.005 [-0.029;0.019]	-0.007 [-0.02;0.002]	-0.011 [-0.029;0.019]	0.019 [-0.005;0.048]
**FS**	-0.003 [-0.022;0.006]	-0.009 [-0.026;0.003]	**0.02 [0.011;0.036]**	0.002 [-0.013;0.018]	0.009 [0.003;0.02]	0.003 [-0.014;0.018]	-0.012 [-0.034;0.001]
**FB**	-0.019 [-0.044;0.005]	-0.005 [-0.029;0.019]	0.002 [-0.013;0.018]	**0.067 [0.033;0.108]**	-0.003 [-0.016;0.009]	0.026 [0.001;0.066]	-0.003 [-0.037;0.022]
**BS**	-0.001 [-0.013;0.008]	-0.007 [-0.02;0.002]	0.009 [0.003;0.02]	-0.003 [-0.016;0.009]	**0.012 [0.006;0.021]**	0.004 [-0.01;0.014]	-0.01 [-0.029;-0.001]
**BF**	0.002 [-0.021;0.028]	-0.011 [-0.029;0.019]	0.003 [-0.014;0.018]	0.026 [0.001;0.066]	0.004 [-0.01;0.014]	**0.062 [0.029;0.106]**	-0.007 [-0.037;0.022]
**Size**	0.012 [-0.012;0.041]	0.019 [-0.005;0.048]	-0.012 [-0.034;0.001]	-0.003 [-0.037;0.022]	-0.01 [-0.029;-0.001]	-0.007 [-0.037;0.022]	**0.063 [0.028;0.113]**

GA450 in low salt

	SF	SB	FS	FB	BS	BF	Size
**SF**	**0.038 [0.017;0.079]**	0.019 [0.001;0.059]	-0.005 [-0.03;0.009]	-0.01 [-0.055;0.041]	-0.001 [-0.021;0.011]	0 [-0.036;0.057]	0.009 [-0.033;0.062]
**SB**	0.019 [0.001;0.059]	**0.038 [0.02;0.087]**	-0.002 [-0.029;0.012]	0.011 [-0.04;0.06]	-0.003 [-0.024;0.01]	0.007 [-0.027;0.071]	0.009 [-0.044;0.051]
**FS**	-0.005 [-0.03;0.009]	-0.002 [-0.029;0.012]	**0.019 [0.01;0.046]**	-0.004 [-0.041;0.026]	0.011 [0.001;0.028]	-0.013 [-0.054;0.015]	-0.017 [-0.055;0.013]
**FB**	-0.01 [-0.055;0.041]	0.011 [-0.04;0.06]	-0.004 [-0.041;0.026]	**0.143 [0.076;0.273]**	-0.018 [-0.051;0.007]	0.099 [0.033;0.21]	-0.075 [-0.163;-0.008]
**BS**	-0.001 [-0.021;0.011]	-0.003 [-0.024;0.01]	0.011 [0.001;0.028]	-0.018 [-0.051;0.007]	**0.013 [0.006;0.032]**	-0.021 [-0.057;0.003]	-0.004 [-0.038;0.016]
**BF**	0 [-0.036;0.057]	0.007 [-0.027;0.071]	-0.013 [-0.054;0.015]	0.099 [0.033;0.21]	-0.021 [-0.057;0.003]	**0.136 [0.058;0.257]**	-0.057 [-0.137;0.013]
**Size**	0.009 [-0.033;0.062]	0.009 [-0.044;0.051]	-0.017 [-0.055;0.013]	-0.075 [-0.163;-0.008]	-0.004 [-0.038;0.016]	-0.057 [-0.137;0.013]	**0.156 [0.075;0.27]**

GA450 in high salt

	SF	SB	FS	FB	BS	BF	Size
**SF**	**0.082 [0.04;0.147]**	0.05 [0.018;0.116]	-0.024 [-0.058;-0.002]	-0.055 [-0.122;-0.013]	-0.007 [-0.029;0.01]	-0.022 [-0.07;0.017]	0.044 [0.007;0.094]
**SB**	0.05 [0.018;0.116]	**0.084 [0.036;0.147]**	-0.025 [-0.062;-0.005]	-0.037 [-0.099;0.008]	-0.013 [-0.039;0.001]	-0.025 [-0.074;0.013]	0.032 [0;0.085]
**FS**	-0.024 [-0.058;-0.002]	-0.025 [-0.062;-0.005]	**0.029 [0.015;0.06]**	0.02 [-0.005;0.067]	0.009 [0.001;0.029]	0.013 [-0.008;0.05]	-0.019 [-0.055;-0.002]
**FB**	-0.055 [-0.122;-0.013]	-0.037 [-0.099;0.008]	0.02 [-0.005;0.067]	**0.128 [0.068;0.242]**	0.003 [-0.021;0.031]	0.057 [0.014;0.142]	-0.043 [-0.102;-0.001]
**BS**	-0.007 [-0.029;0.01]	-0.013 [-0.039;0.001]	0.009 [0.001;0.029]	0.003 [-0.021;0.031]	**0.014 [0.008;0.03]**	0.006 [-0.013;0.028]	-0.009 [-0.032;0.005]
**BF**	-0.022 [-0.07;0.017]	-0.025 [-0.074;0.013]	0.013 [-0.008;0.05]	0.057 [0.014;0.142]	0.006 [-0.013;0.028]	**0.087 [0.044;0.168]**	-0.015 [-0.06;0.02]
**Size**	0.044 [0.007;0.094]	0.032 [0;0.085]	-0.019 [-0.055;-0.002]	-0.043 [-0.102;-0.001]	-0.009 [-0.032;0.005]	-0.015 [-0.06;0.02]	**0.061 [0.027;0.121]**

Raw output from R is available at: https://github.com/ExpEvolWormLab/Mallard_Robertson/tree/main/output_files/txt/output_files/G_mat_tables/
