# Supplementary material for: Selection and the direction of phenotypic evolution
Source: eLife. 2023 Aug 31;12:e80993. doi: 10.7554/eLife.80993 (PMC10564456; doi:10.7554/eLife.80993)
Supplement: Figure 7—figure supplement 1—source data 1. — See table. [file elife-80993-fig7-figsupp1-data1.pdf]

	**G**A150 - Low Salt							**G**A150 - High Salt						
	g_{max}	g_2	g_3	g_4	g_5	g_6	g7	g_{max}	g_2	g_3	g_4	g_5	g_6	g7
Eigenvalues	0.157	0.07	0.036	0.019	0.011	0.008	0.003	0.147	0.088	0.035	0.02	0.01	0.007	0.004
HPD lower	0.085	0.039	0.023	0.013	0.008	0.005	0.002	0.086	0.046	0.023	0.014	0.008	0.005	0.003
HPD upper	0.294	0.129	0.062	0.029	0.017	0.011	0.005	0.254	0.136	0.056	0.034	0.017	0.01	0.006
Proportion	0.516	0.23	0.118	0.062	0.036	0.026	0.01	0.473	0.283	0.113	0.064	0.032	0.023	0.013
Trait loadings:														
SF	0.034	-0.529	-0.307	-0.19	-0.411	0.645	-0.056	-0.363	-0.338	0.162	0.491	-0.472	0.45	0.247
SB	-0.013	-0.663	-0.398	0.251	0.146	-0.56	0.066	-0.236	-0.568	0.517	-0.055	0.098	-0.569	-0.13
FS	0.096	0.122	0.03	-0.077	-0.714	-0.317	0.6	0.132	0.18	-0.098	-0.11	-0.629	-0.535	0.497
FB	-0.659	-0.141	0.368	0.59	-0.215	0.13	0.005	0.676	-0.484	0.117	-0.372	-0.243	0.311	-0.037
BS	0.029	0.113	-0.054	-0.011	-0.5	-0.325	-0.792	0.06	0.123	-0.079	0.214	-0.499	-0.141	-0.812
BF	-0.578	-0.19	0.189	-0.738	0.066	-0.211	-0.006	0.295	-0.419	-0.58	0.532	0.215	-0.26	0.068
Size	0.47	-0.444	0.757	-0.045	-0.009	-0.054	-0.068	-0.498	-0.326	-0.583	-0.526	-0.133	0.05	-0.095

	**G**A250 - Low Salt							**G**A250 - High Salt						
	g_{max}	g_2	g_3	g_4	g_5	g_6	g7	g_{max}	g_2	g_3	g_4	g_5	g_6	g7
Eigenvalues	0.183	0.088	0.04	0.023	0.011	0.007	0.003	0.121	0.069	0.04	0.023	0.014	0.007	0.004
HPD lower	0.106	0.047	0.025	0.015	0.007	0.005	0.002	0.078	0.041	0.026	0.015	0.008	0.005	0.003
HPD upper	0.288	0.157	0.067	0.035	0.018	0.01	0.005	0.22	0.118	0.064	0.038	0.021	0.011	0.006
Proportion	0.515	0.248	0.113	0.065	0.031	0.02	0.008	0.435	0.248	0.144	0.083	0.05	0.025	0.014
Trait loadings:														
SF	-0.374	-0.355	0.461	-0.136	-0.077	0.64	0.296	-0.27	0.113	-0.509	-0.351	0.228	0.688	0.08
SB	-0.459	-0.375	0.417	0.162	0.043	-0.599	-0.297	-0.32	0.293	0.022	-0.694	0.14	-0.556	-0.015
FS	0.086	0.149	0.229	0.323	0.755	-0.097	0.484	0.142	-0.201	-0.057	0.112	0.82	-0.11	-0.488
FB	0.206	-0.533	-0.282	0.74	-0.139	0.162	0.016	0.585	0.489	0.469	-0.247	0.194	0.293	0.118
BS	0.095	0.096	0.163	0.108	0.421	0.42	-0.768	0.091	-0.185	-0.12	0.101	0.399	-0.203	0.856
BF	0.178	-0.632	-0.32	-0.531	0.424	-0.074	-0.008	0.527	0.33	-0.708	0.065	-0.156	-0.276	-0.084
Size	-0.748	0.139	-0.593	0.097	0.215	0.117	-0.005	-0.419	0.692	0.031	0.554	0.185	-0.038	0.037

	**G**A450 - Low Salt							**G**A450 - High Salt						
	g_{max}	g_2	g_3	g_4	g_5	g_6	g7	g_{max}	g_2	g_3	g_4	g_5	g_6	g7
Eigenvalues	0.314	0.097	0.052	0.032	0.016	0.011	0.005	0.263	0.084	0.043	0.029	0.016	0.012	0.006
HPD lower	0.174	0.053	0.03	0.02	0.011	0.007	0.003	0.153	0.048	0.029	0.018	0.011	0.007	0.004
HPD upper	0.577	0.219	0.089	0.046	0.025	0.017	0.008	0.526	0.165	0.074	0.044	0.025	0.016	0.009
Proportion	0.596	0.184	0.099	0.061	0.03	0.021	0.009	0.581	0.185	0.095	0.064	0.035	0.026	0.013
Trait loadings:														
SF	-0.038	-0.144	0.72	-0.14	0.44	0.493	-0.061	-0.453	0.368	0.166	0.037	0.782	0.027	-0.134
SB	0.021	-0.227	0.634	0.307	-0.461	-0.485	0.061	-0.4	0.453	-0.525	-0.464	-0.306	0.21	0.08
FS	-0.005	0.248	0.036	0.158	-0.572	0.503	-0.576	0.193	-0.164	0.13	-0.2	0.077	0.858	-0.371
FB	0.619	-0.233	-0.138	0.677	0.194	0.207	0.06	0.593	0.452	-0.475	0.389	0.22	0.124	0.064
BS	-0.072	0.201	0.041	0.043	-0.348	0.416	0.811	0.069	-0.097	0.142	-0.173	0.21	0.275	0.903
BF	0.565	-0.442	-0.043	-0.615	-0.312	0.084	0.017	0.355	0.59	0.592	-0.348	-0.183	-0.139	-0.039
Size	-0.539	-0.759	-0.236	0.148	-0.102	0.213	-0.009	-0.34	0.267	0.289	0.663	-0.404	0.33	0.13

Raw output from R is available at: https://github.com/ExpEvolWormLab/Mallard_Robertson/tree/main/output_files/txt/output_files/G_matrix_eigendecomposition/
