# Supplementary material for: Selection and the direction of phenotypic evolution
Source: eLife. 2023 Aug 31;12:e80993. doi: 10.7554/eLife.80993 (PMC10564456; doi:10.7554/eLife.80993)
Supplement: Figure 8—source data 1. [file elife-80993-fig8-data1.pdf]

Figure 8A:

Null

95% CI	[0.80 - 0.91]

Observed

Mean	0.93
83% CI	[0.89 - 0.97]
95% CI	[0.91 - 0.96]

Figure 8B:

Null

83% CI	[60.0 - 90.0]
95% CI	[45.6 - 90.0]

Observed

	gmax	g2	g3
Mean	15.4	85.0	81.6
83% CI	[11.7 - 18.6]	[81.5 - 90.0]	[75.9 - 88.3]
95% CI	[10.8 - 20.3]	[78.0 - 90.0]	[74.9 - 90.0]

Figure 8C:

Null

95% CI	[0.87 - 0.96]

Observed

Mean	0.94
83% CI	[0.92 - 0.96]
95% CI	[0.90 - 0.97]

Figure 8D:

Null

83% CI	[57.2 - 90.0]
95% CI	[43.1 - 90.0]

Observed

	gmax	g2	g3
Mean	14.7	86.5	84.4
83% CI	[11.7 - 17.3]	[83.9 - 90.0]	[80.5 - 89.9]
95% CI	[11.0 - 19.3]	[81.7 - 90.0]	[78.2 - 90.0]
