# Supplementary material for: Selection and the direction of phenotypic evolution
Source: eLife. 2023 Aug 31;12:e80993. doi: 10.7554/eLife.80993 (PMC10564456; doi:10.7554/eLife.80993)
Supplement: Figure 9—source data 2. [file elife-80993-fig9-data2.pdf]

**GA150**

| | **β** | | | |
|---|---|---|---|---|
| | 83% CI | | 95% CI | |
| **Trait** | lower | upper | lower | upper |
| SF | -2.97 | -1.14 | -3.54 | -0.69 |
| SB | -0.77 | 0.72 | -1.16 | 1.14 |
| FS | -1.5 | 0.62 | -2.18 | 1.21 |
| FB | -1.16 | 0.14 | -1.56 | 0.45 |
| BS | -3.31 | -0.28 | -4.23 | 0.49 |
| BF | -3.04 | -1.55 | -3.59 | -1.25 |
| Size | -2.25 | -0.57 | -2.92 | -0.28 |

**GA250**

| | **β** | | | |
|---|---|---|---|---|
| | 83% CI | | 95% CI | |
| **Trait** | lower | upper | lower | upper |
| SF | -2.17 | -0.54 | -2.6 | -0.06 |
| SB | 0.99 | 2.49 | 0.67 | 2.97 |
| FS | 2.05 | 4.2 | 1.42 | 4.7 |
| FB | -3.01 | -1.67 | -3.35 | -1.33 |
| BS | -4.08 | -1.09 | -5.19 | -0.46 |
| BF | -0.21 | 1.08 | -0.63 | 1.39 |
| Size | -0.06 | 1.44 | -0.55 | 1.78 |

**GA450**

| | **β** | | | |
|---|---|---|---|---|
| | 83% CI | | 95% CI | |
| **Trait** | lower | upper | lower | upper |
| SF | -4.18 | -2.29 | -4.73 | -1.82 |
| SB | 1.49 | 3.08 | 1.07 | 3.57 |
| FS | -0.32 | 1.81 | -0.94 | 2.41 |
| FB | -0.7 | 0.58 | -1.04 | 0.96 |
| BS | -5.98 | -2.7 | -6.97 | -1.96 |
| BF | -1.64 | -0.28 | -2.06 | 0.1 |
| Size | -1.93 | -0.32 | -2.59 | -0.01 |

**Raw output from R is available at:**
https://github.com/ExpEvolWormLab/Mallard_Robertson/blob/main/output_files/txt/Selection_gradients.txt
